# Low *Agrobacterium tumefaciens* inoculum levels and a long co-culture period lead to reduced plant defense responses and increase transgenic shoot production of sunflower (*Helianthus annuus* L.)

**DOI:** 10.1007/s11627-016-9774-5

**Published:** 2016-07-12

**Authors:** Zhifen Zhang, John J. Finer

**Affiliations:** 1 Department of Horticulture and Crop Science, OARDC/The Ohio State University, 1680 Madison Avenue, Wooster, OH 44691 USA; 2 Department of Horticulture, The University of Georgia Tifton Campus, Tifton, GA 31793 USA

**Keywords:** *Agrobacterium*, Inoculum density, Co-culture time, Shoot organogenesis, Plant defense

## Abstract

*Agrobacterium*-mediated plant transformation is typically conducted by inoculating plant tissues with an *Agrobacterium* suspension containing approximately 10^8^–10^9^ bacteria mL^−1^, followed by a 2–3-d co-culture period. Use of longer co-culture periods could potentially increase transformation efficiencies by allowing more time for *Agrobacterium* to interact with plant cells, but bacterial overgrowth is likely to occur, leading to severe tissue browning and reduced transformation and regeneration. Low bacterial inoculum levels were therefore evaluated as a means to reduce the negative outcomes associated with long co-culture. The use of low inoculum bacterial suspensions (approximately 6 × 10^2^ bacteria mL^−1^) followed by long co-culture (15 d) led to the production of an average of three transformed sunflower shoots per explant while the use of high inoculum (approximately 6 × 10^8^ bacteria mL^−1^) followed by short co-culture (3 d) led to no transformed shoots. Low inoculum and long co-culture acted synergistically, and both were required for the improvement of sunflower transformation. Gene expression analysis via qRT-PCR showed that genes related to plant defense response were generally expressed at lower levels in the explants treated with low inoculum than those treated with high inoculum during 15 d of co-culture, suggesting that low inoculum reduced the induction of plant defense responses. The use of low inoculum with long co-culture (LI/LC) led to large increases in sunflower transformation efficiency. This method has great potential for improving transformation efficiencies and expanding the types of target tissues amenable for transformation of different plant species.

## Introduction

Since the first reports of using *Agrobacterium tumefaciens* to introduce genes into plant cells (Bevan *et al.*^1983^; Fraley *et al.*^1983^; Herrera-Estrella *et al.*^1983^), *Agrobacterium*-mediated transformation has become the method of choice for gene introduction in most plant species (Fillatti *et al.*^1987^; Bidney *et al.*^1992^; Perl *et al.*^1996^; Trick and Finer 1997; Bond and Roose 1998; Clough and Bent 1998; Zhao *et al.*^2002^; Cheng *et al.*^2003^, ^2004^). With a more thorough understanding of how *A. tumefaciens* delivers transfer DNA (T-DNA) into plant cells and integrates it into plant genome (Gelvin 2003, 2012), *Agrobacterium*-mediated plant transformation has been continuously improved by optimizing conditions for virulence gene induction (Alt-Mörbe *et al.*^1989^; Godwin *et al.*^1991^), developing high-virulence bacterial strains (Hood *et al.*^1993^; Hansen *et al.*^1994^), identifying crop-specific strains (Benzle *et al.*^2015^), adopting efficient inoculation methods (Bidney *et al.*^1992^; Trick and Finer 1997), and reducing plant defense responses (Perl *et al.*^1996^; Olhoft and Somers 2001). Although different protocols have been developed for transformation of many plant species, plant tissues are always inoculated with an *A. tumefaciens* suspension containing millions of bacteria (Fillatti *et al.*^1987^; Hiei *et al.*^1994^; Bond and Roose 1998; Zhao *et al.*^2002^). This approach is likely based on the predominant conception that the highest transformation rates result from the use of large numbers of bacteria to infect a large number of plant cells (Cheng *et al.*^2004^).

When plant tissues are inoculated with *A. tumefaciens*, the presence of this plant pathogen can be detected by the plant defense system, inducing responses that may limit transformation and regeneration from transformed cells. Recognition of the pathogen-associated molecular pattern (PAMP), EF-Tu, from *A. tumefaciens*, by a plant kinase receptor (EFR) in *Arabidopsis thaliana*, reduced transformation by *A. tumefaciens* through PAMP-triggered immunity (PTI) responses (Zipfel *et al.*^2006^). Additionally, induction of several plant defense genes was observed following inoculation of *A. tumefaciens* onto either *A. thaliana* cell cultures (Ditt *et al.*^2006^), *A. thaliana* inflorescence stalks (Lee *et al.*^2009^), tobacco cell cultures (*Nicotiana tabacum*, Veena *et al.*^2003^), or wheat (*Triticum aestivum*) embryogenic calluses (Zhou *et al.*^2013^). If pathogen challenges continued, the amplitude of defense responses further increased, leading to programmed cell death (PCD) or hypersensitive response (HR) (Jones and Dangl 2006; Coll *et al.*^2011^). Tissue browning, observed during *Agrobacterium*-mediated plant transformation, was commonly associated with HR (Perl *et al.*^1996^; Hansen 2000; Olhoft and Somers 2001).

Considering that plant defense responses triggered by *A. tumefaciens* can reduce plant transformation efficiency, a reduction of plant defense activation could potentially improve transformation rates. The use of antioxidants to suppress the oxidative burst, a common event at the early stage of HR (Lamb and Dixon 1997), has led to improvements in transformation of maize (*Zea mays*, Frame *et al.*^2002^), soybean (*Glycine max*, Olhoft and Somers 2001), and grape (*Vitis vinifera*, Perl *et al.*^1996^). Increases in transformation efficiency were also observed in the *efr* mutant of *A. thaliana* that lost the ability to recognize EF-Tu (Zipfel *et al.*^2006^). The activation of plant defenses could depend on the inoculum density, as a threshold inoculum density of *Pseudomonas fluorescens* was required for the induction of systemic resistance in radish (*Raphanus sativus*, Leeman *et al.*^1995^; Raaijmakers *et al.*^1995^). Since induction of plant defense genes could result following inoculation of plant tissues with a high number of infecting cells, the use of low-density inoculum may enable *A. tumefaciens* to evade the host detection by not activating plant defense responses. This may be the likely scenario in field infestations, where low numbers of this bacterium found in the soil (Benzle *et al.*^2015^) infect susceptible plants at wound sites.

Co-culture is critical for plant transformation because it is the period when *A. tumefaciens* cells interact with host cells and deliver the processed T-DNA into the targeted cells. Modification of co-culture conditions through medium modification or plant tissue preparation has been explored to increase transformation efficiency (Santarém *et al.*^1998^; Olhoft and Somers 2001; Cheng *et al.*^2003^). Regardless of the modifications, co-culture periods for most transformation protocols were limited to 2–3 d (Bidney *et al.*^1992^; Perl *et al.*^1996^; Trick and Finer 1997; Olhoft and Somers 2001; Cheng *et al.*^2003^). Although an increase in the number of transformed cells was observed with 5–6 d co-culture periods in citrange (*Citrus sinensis* × *Poncirus trifoliata*, Cervera *et al.*^1998^), rice (*Oryza sativa*, Rashid *et al.*^1996^), and sunflower (*Helianthus annuus*, Sujatha *et al.*^2012^), extensions of co-culture periods were not adopted due to severe bacterial overgrowth that suppressed plant regeneration. To prevent *A. tumefaciens* overgrowth during co-culture, approaches that could slow bacterial growth have been evaluated, such as placement of plant tissues on filter paper (Ozawa 2009), addition of silver nitrate to media (Zhao *et al.*^2002^), and desiccation of explants (Cheng *et al.*^2003^). With most of these approaches, the co-culture time remained less than 4 d, suggesting that slowing bacterial growth could not completely preclude unwanted damages to plant tissues associated with long co-culture. Although a 7-d co-culture was used to improve transformation in rubber tree (*Hevea brasiliensis*), a low co-culture temperature was used, which slowed the growth of the bacteria (Blanc *et al.*^2006^).

Although sunflower tissues seem to be quite responsive to *Agrobacterium*-mediated transformation (de Ropp 1947; Murai *et al.*^1983^), sunflower regeneration systems are inefficient and the generation of transgenic plants remains problematic. Transgenic sunflower plants were first obtained by using *Agrobacterium*-mediated transformation followed by shoot organogenesis from callus induced from hypocotyl tissue (Everett *et al.*^1987^). Since shoot regeneration from callus was not consistent, different transformation protocols were developed using target tissues that were more reliable for plant recovery. Most of the sunflower transformation protocols targeted the shoot apex (Bidney *et al.*^1992^; Weber *et al.*^2003^) or embryo axis (Grayburn and Vick 1995), but the frequency of transgenic shoot production has remained low due to the low shooting response from these tissues. Cotyledon tissues of dry seeds gave a high shoot induction response (Power 1987), and that tissue was also shown to be suitable for sunflower transformation (Sujatha *et al.*^2012^). Unfortunately, the number of transgenic shoots obtained from each explant was not reported (Sujatha *et al.*^2012^), making a comparison of efficiency with previous studies difficult. In that report, a short increase in co-culture time by 2–4 d did not lead to any increase in transgenic shoot production, and a 2-d co-culture period was recommended (Sujatha *et al.*^2012^).

In this study, a simple and straightforward method is presented for significantly improving transformation rates using a low-density inoculum of *Agrobacterium* followed by a long co-culture period using sunflower as a model. The use of low inoculum/long co-culture (LI/LC) led to a significant increase in transgenic shoot production that has never been seen with the traditional inoculum and co-culture protocols for plant transformation using *Agrobacterium* in sunflower.

## Materials and Methods

### **Plant material preparation**

Seeds of sunflower (*H. annuus* L.) RHA280 were harvested from plants grown under greenhouse conditions as previously described (Zhang and Finer 2015), and stored in sealed plastic bags at 4°C in the dark for up to 1 yr. After pericarps were removed manually, high-quality kernels (those without developmental defects or necrotic regions) were used for transformation. Kernels were surfaced sterilized with 5% (*v*/*v*) commercial bleach (8.25% [*w*/*v*] sodium hypochlorite; Clorox, Oakland, CA) for 20 min, and rinsed with sterilized distilled water 8–10 times. After the embryo axis was removed by making a cut 1–2 mm from the cotyledonary node perpendicular to the longitudinal axis, the remaining cotyledons were immersed in liquid shoot induction medium (SIM). After overnight immersion, the seed coat was easily removed from the cotyledons with minimal damage to cotyledon tissues. SIM was composed of Murashige and Skoog salts (Murashige and Skoog 1962), Gamborg’s B5 vitamins (Gamborg *et al.*^1968^), 30 g L^−1^ sucrose (MP Biomedicals, Solon, OH), 1.5 mg L^−1^ 6-benzylaminopurine, and 0.2 mg L^−1^ 1-naphthaleneacetic acid. The medium pH was adjusted to 5.7, and sterilized by autoclaving at 121°C for 20 min. All medium components were obtained from Sigma-Aldrich® (St Louis, MO) if not otherwise specified.

### **Agrobacterium strain and binary vector**

*A. tumefaciens* strain EHA105 was used for plant transformation. An expression cassette, composed of a sunflower *polyubiquitin* gene promoter (*HaUbi*, GenBank accession KX231815) cloned from RHA280, a soluble *green fluorescent protein* (*gfp*) gene coding sequence, and a *nopaline synthase* (*nos*) terminator, was inserted into the multiple cloning site of the pCAMBIA1300 binary vector (CAMBIA, Canberra, Australia), upstream of the *hygromycin phosphotransferase* (*hptII*) gene regulated by the cauliflower mosaic virus (CaMV) 35S promoter and CaMV 35S terminator. The binary vector was introduced into EHA105 competent cells by the freeze–thaw approach (Chen *et al.*^1994^). Bacteria were then grown on a modified yeast extract peptone (YEP) medium (pH 7.0) at 28°C for 2 d. YEP was composed of 5 g L^−1^ yeast extract (Thermo-Fisher Scientific, Waltham, MA), 10 g L^−1^ Bacto™ peptone (Becton, Dickinson & Company, Sparks, MD), 0.5 g L^−1^ MgSO_4_•7HO_2_, 1 g L^−1^ sucrose, solidified with 20 g L^−1^ Bacto™ agar (Becton, Dickinson & Company), and 100 mg L^−1^ filter-sterilized kanamycin (Thermo-Fisher Scientific). Bacterial colonies were screened for the presence of the introduced plasmid by polymerase chain reaction (PCR) using specific primers for *HaUbi*:*gfp* and *virG* genes (Table 1) as previously described (Benzle *et al.*^2015^). PCR products were electrophoresed in a 1% (*w*/*v*) agarose gel stained with ethidium bromide and visualized under UV (*λ* = 365 nm) illumination. A glycerol stock (containing 15% [*v*/*v*] sterilized glycerol) was made for overnight liquid culture from a PCR-positive colony and stored at −80°C.

**Table 1.** Primer sequences for amplification of the *HaUbi* promoter, *gfp*, and *virG*

Primer name	Primer sequences
HaUbiF	CGCAGTAGTTTGAAAGTAACCC
HaUbiR	CAAACAGATTAATACCCTAAGC
gfpR	CCGTAGGTGGCATCGC
VirGF	ATCTYAATTTRGGKCGYGAAGA
VirGR	CACRTCMGCGTCRAAGAAATA

### **Bacterial inoculum and plant tissue transformation**

Bacterial cultures were initiated from the glycerol stock, grown on solid YEP medium containing 100 mg L^−1^ kanamycin at 25°C, and maintained for up to 1 mo. For each experiment, a new single colony was inoculated into 2 mL liquid YEP medium containing 100 mg L^−1^ kanamycin, and incubated in the dark at 28°C at 150 rpm. After 24 h, 500 μL of the culture was inoculated into 50 mL liquid YEP containing 100 mg L^−1^ kanamycin, and incubated in the dark at 28°C at 150 rpm overnight. After the optical density at *λ* = 600 nm (OD_600_) of the culture reached between 0.6 and 1.0, the bacterial culture was centrifuged at 3000×*g* for 10 min at 4°C. The pellet was re-suspended in inoculation medium, consisting of liquid SIM supplemented with 100 μM acetosyringone (1 M stock solution in dimethyl sulfoxide, filter-sterilized; PhytoTechnology Laboratories®, Overland Park, KS) and 0.02% (*v*/*v*) Silwet-77 (Lehle Seeds, Round Rock, TX), with OD_600_ adjusted to about 0.55 (10^8^–10^9^ bacteria mL^−1^). The *Agrobacterium* inoculum was incubated at 25°C without shaking in a laminar flow hood for about 4 h before use in plant transformation experiments.

Sunflower cotyledons were inoculated with *A. tumefaciens* by immersing them in the bacterial suspension for 10 min, and then blotting them on filter paper. Three additional cuts were made on each cotyledon with 1–2 mm between each cut, parallel to the first cut and perpendicular to the longitudinal axis, on filter paper wetted with the bacterial suspension, producing three cotyledonary explants having two cut sides, with the distal round end discarded. Cotyledon explants were placed on SIM solidified with 0.2% (*w*/*v*) Gelrite™ (Research Products International, Mt. Prospect, IL), generally with a cut side close to proximal end in contact with the medium, and incubated under standard culture conditions of 25°C with a 16 h photoperiod (40 μmol m^−2^ s^−1^) using Plant & Aquarium fluorescent lamps (Philips Lighting, Somerset, NJ) alternating with Gro-Lux® wide spectrum fluorescent lamps (Sylvania®, Mississauga, Canada). After 3 d of co-culture, the explants were washed with liquid SIM containing 400 mg L^−1^ Timentin® (SmithKline Beecham Pharmaceuticals, Philadelphia, PA), blotted dry, and transferred to solid SIM containing 400 mg L^−1^ Timentin and 7.5 mg L^−1^ hygromycin B (Calbiochem, La Jolla, CA).

### **Evaluation of inoculum density and co**-**culture time on transformation**

Different inoculum densities were first evaluated using a 15-d co-culture period. Log10 serial dilutions of a high-density *A. tumefaciens* suspension (OD_600_ ≈ 0.55), obtained as previously described, were made using inoculation medium as the diluent. Fifteen cotyledons were immersed in 9 mL of either an undiluted *A. tumefaciens* suspension, or one of the 10^−2^, 10^−4^, 10^−6^, 10^−8^, or 10^−10^ dilutions, for 10 min. The cotyledon explants were prepared and plated on solidified SIM as previously described. Cotyledon explants were co-cultured with *A. tumefaciens* for 15 d under standard culture conditions. To calculate the number of viable bacteria in the suspensions, 100 μL each of the 10^−5^ and 10^−6^ dilutions was plated on solid YEP medium containing 100 mg L^−1^ kanamycin and incubated at 25°C for 3 d. For each experiment, the concentration of bacteria in the 10^−5^ and 10^−6^ dilutions was used to calculate the number of colony-forming units (CFU) mL^−1^ in each different inoculum.

To determine the effects of low and high inoculum density using short and long co-culture times, sunflower cotyledon tissues were inoculated with either undiluted *A. tumefaciens* suspension (referred to as high inoculum hereinafter) or the 10^−6^ dilution (referred to as low inoculum hereinafter), followed by either a co-culture period of 3 d (referred to as short co-culture hereinafter) or a co-culture period of 15 d (referred to as long co-culture hereinafter). The explants with short co-culture were washed with liquid SIM containing 400 mg L^−1^ Timentin® after a 3-d co-culture period, and transferred to solid SIM containing 400 mg L^−1^ Timentin® for further culture, while the explants with long co-culture were maintained on SIM for 15 d without interruption. Hygromycin selection was not applied in any of these four treatments. In addition, the transformation approach using high inoculum with short co-culture followed by selection with 7.5 mg L^−1^ hygromycin B (referred to as high inoculum/short co-culture plus Hyg-selection hereinafter) was included.

### **Observation of GFP in plant tissue**

GFP expression in sunflower tissues was monitored using a MZFLIII stereomicroscope (Leica, Heerbrugg, Switzerland) equipped with a GFP-2 filter set (excitation 480 ± 40 nm; emission 510 nm) and a pE-100 light-emitting diode (Andover, Hampshire, UK) as an excitation light source. To gauge the transformation efficiency, the number of adventitious shoots expressing GFP (“GFP shoots”) for each explant was counted 15 d after inoculation. Shoots with only dispersed GFP-expressing cells that did not form a solid sector were not counted as GFP shoots. Images of explants were collected with a Nikon (Melville, NY) Coolpix 990 digital camera mounted on the MZFLIII fluorescence stereomicroscope. The total number of adventitious shoots was also counted for each explant in order to measure the effects of treatments on the efficiency of shoot induction.

### **Detection of*Agrobacterium*on explants**

Cotyledons were inoculated with low or high inoculum, followed by long co-culture on SIM as previously described. At 0, 3, 6, 9, 12, and 15 d after inoculation, seven cotyledon explants were removed from culture for each treatment, and each explant was individually placed in a 1.5 mL microfuge tube containing 100 μL liquid YEP. The cotyledon tissues were homogenized using sterilized plastic pestles (Argos Technologies, Elgin, IL) driven by an electric power drill. Homogenates of 100 μL for each sample were plated on YEP medium containing 100 mg L^−1^ kanamycin. After incubation for 3 d at 25°C, the presence of *Agrobacterium* on explants was determined based on the growth of bacteria on YEP.

### **Quantitative real**-**time for selected genes after*Agrobacterium*inoculation**

Upregulated genes, associated with plant defense response to *A. tumefaciens* infection, were selected from studies of *Agrobacterium* inoculation of *A. thaliana* cell cultures (Ditt *et al.*^2006^), inflorescence stalks (Lee *et al.*^2009^), tobacco cell cultures (Veena *et al.*^2003^), and wheat embryogenic calluses (Zhou *et al.*^2013^). Orthologs of the selected genes were identified in the sunflower genome by running basic local alignment search tool (BLAST) using the HeliaGene database (www.heliagene.org/HA412.v1.1.bronze.20141015) with the amino acid sequences of the selected *A. thaliana* genes (Table 2), and the best hit with the highest percentage of identity and the lowest expectation value was chosen for each gene. Afterwards, the amino acid sequence of the best hit for each sunflower gene was used to run BLAST using The Arabidopsis Information Resource (TAIR; www.arabidopsis.org) database to confirm that the selected sunflower gene and the best hit of *A. thaliana* gene belonged to the same gene family. The selected genes were *HaPR1*, *HaPR2*, *HaMBL*, *HaWRKY53*, and *HaOxo* (Table 2). In addition, a sunflower ortholog of the *Arabidopsis shoot meristemless* (*STM*) gene (*HaSTM*) was identified and included in this study to monitor how shoot induction was influenced by different inoculation methods (Table 2). Specific primers (Table 3) for qRT-PCR were then designed using the real-time qPCR Assay Tool (Integrated DNA Technologies, Coralville, IA) or the PrimerQuest® Tool (Integrated DNA Technologies).

**Table 2.** Sunflower orthologs studied by quantitative RT-PCR analysis and the three reference genes

Sunflower orthologs	HeliaGene database gene identifiers	Putative function	TAIR: *Arabidopsis* gene identifiers
*HaPR1*	Ha412v1r1_12g021430	Pathogenesis-related protein 1/Allergen V5/Tpx-1-related	At2g19970
*HaPR2*	Ha412v1r1_14g008310	Pathogenesis-related protein 2/Glycoside hydrolase	At2g43610
*HaMBL*	Ha412v1r1_11g021150	Mannose-binding lectin/Bulb-type lectin/S-locus glycoprotein	At1g78850
*HaWRKY53*	Ha412v1r1_00g070960	*WRKY53* transcription factor	At4g23810
*HaOxo*	Ha412v1r1_13g029660	Oxoglutarate/Iron-dependent dioxygenase	At3g13610
*HaSTM*	Ha412v1r1_15g010080	Homeodomain transcription factor/Shoot meristemless/*KNOX* family	At1g62360
Reference genes			
*HaActin*	Ha412v1r1_17g024510	Actin cross-linking	At1g27100
*HaRPS2*	Ha412v1r1_12g009050	Ribosomal protein S2	At3g04770
*HaEFh*	Ha412v1r1_03g045600	Elongation factor hand domain pair	At3g43810

**Table 3.** Primer sequences for quantitative real-time PCR

Gene names	Forward primer	Reverse primer	Expected amplicon size (bp)
*HaPR1*	GGTGGACCTTATGGTGAGAAC	CACGTATTGGTAGTGTGGTCATA	113
*HaPR2*	TTGGTTCCGCTAGTTCAGAAG	GGATATTGGGTGTTAAGTTCGTC	148
*HaMBL*	CAAACCCTACATCATCGTCAAATC	CTAAGGTTTCCGTCTATCCCTAATC	83
*HaWRKY53*	CGATGACGGTTATAGTTGGAGG	TCGTCTGTTCTCTGCACTTG	135
*HaOxo*	GAGTGGAAGGATTATCTCAGCC	ACCTCTTGACAACCGTTTCAG	124
*HaSTM*	GTCTCATCCTCATTACCCTCG	CGGAGCGACTGGACATTG	149
*HaActin*	CCCAGTTCTCTTAGGCACAAC	GCCCTCAAATATGTCCCTACG	141
*HaRps2*	GATGGTTTTGCAGATGCGAG	TCCTCTGGTTCCCTGTAGAAG	87
*HaEFh*	GCTTTCAGCCTCTTCGACAAG	CCATCAGCATCCACCTCATTG	134

Cotyledons were inoculated with either low or high inoculum, and explants were prepared and plated on SIM for co-culture as previously described. Explants derived from cotyledons, immersed in inoculation medium without *A. tumefaciens* for 10 min, were used as a non-inoculated control. Seven cotyledon explants were removed from culture at 3 h, or 3, 6, 9, 12, and 15 d after inoculation, frozen in liquid nitrogen, and stored at −80°C. RNA extraction was performed within 1 mo. Three independent experiments were conducted.

Total RNA was isolated by using the RNeasy® Plant Mini Kit (QIAGEN, Hilden, Germany), and genomic DNA was removed using the on-column RNase-Free DNase Set (QIAGEN) according to the manufacturer’s instructions. RNA samples were screened by PCR with *HaOxo* primers (Table 3), which spanned an 887 bp intron, and the detection of a 1011 bp amplicon in PCR products after electrophoresis indicated the presence of genomic DNA contaminant. The samples with detectable genomic DNA contamination were further treated with the Ambion® DNA-Free Removal Kit (Thermo-Fisher Scientific) according to the manufacturer’s instructions until genomic DNA was undetectable. RNA concentration was quantified using a Nanodrop® ND-1000 spectrophotometer (Thermo-Fisher Scientific), and RNA integrity was determined by gel electrophoresis. Single-strand cDNA was synthesized with a RETROscript® Reverse Transcription Kit (Thermo-Fisher Scientific) from 1–2 μg of total RNA according to the manufacturer’s instructions. The products were diluted, and 5 ng cDNA was used as template for qRT-PCR.

Quantitative RT-PCRs were conducted in 20 μL reactions using iQ™ SYBR® Green Supermix (Bio-Rad, Hercules, CA) following the manufacturer’s instructions, and the iQ™5 optical system (Bio-Rad) was used to measure target cDNA levels. The PCR cycling conditions were 3 min at 95°C; 40 cycles of 10 s at 95°C and 30 s at 60°C; and a melt curve profiling program with a constant increase by 0.5°C every 30 s from 55 to 95°C. Each gene assay was conducted three times for each sample. The data (quantification cycle, Cq) were obtained from the iQ™5 optical system software (Bio-Rad), and analyzed according to the qBase relative quantification framework (Hellemans *et al.*^2007^). Amplification efficiency of each assay was estimated based on the qRT-PCR data of a 5-log serial dilution (0.005, 0.05, 0.5, 5, 50 ng μL^−1^) of the pooled cDNA from all samples in each independent experiment. The Cq values were converted into relative quantity and then normalized by three reference genes (Table 2) using the geNorm method (Vandesompele *et al.*^2002^). The reference genes were *HaActin*, *HaRPS2*, and *HaEFh* (Table 2), and the stability of the reference genes was determined by the gene-stability measure (Vandesompele *et al.*^2002^; Hellemans *et al.*^2007^) (*M* = 0.72). The means of normalized relative quantity were calculated from three independent experiments for each gene/treatment/time point.

### **Data analyses**

In a transformation experiment, each treatment consisted of at least 3 Petri dishes, and each Petri dish contained 15 explants from 5 different cotyledons. The experiments were repeated at least three times using a completely randomized design. The data of transformation and shoot organogenesis were analyzed using SAS/STAT software (Version 9.4 of SAS System© 2002–2012, SAS Institute Inc., Cary, NC) with the GLM procedure, and means comparison was conducted using Duncan’s multiple range test (*α* = 0.05). Logarithm transformation of the normalized relative quantity data for gene expression was conducted (Rieu and Powers 2009) before data analysis, and the transformed data were analyzed by the GLM procedure for each gene/time point, followed by mean comparison using Duncan’s multiple range test (*α* = 0.05).

## Results and Discussion

### **Transformation efficiency was low with high inoculum and short co**-**culture**

**Figure 1.**
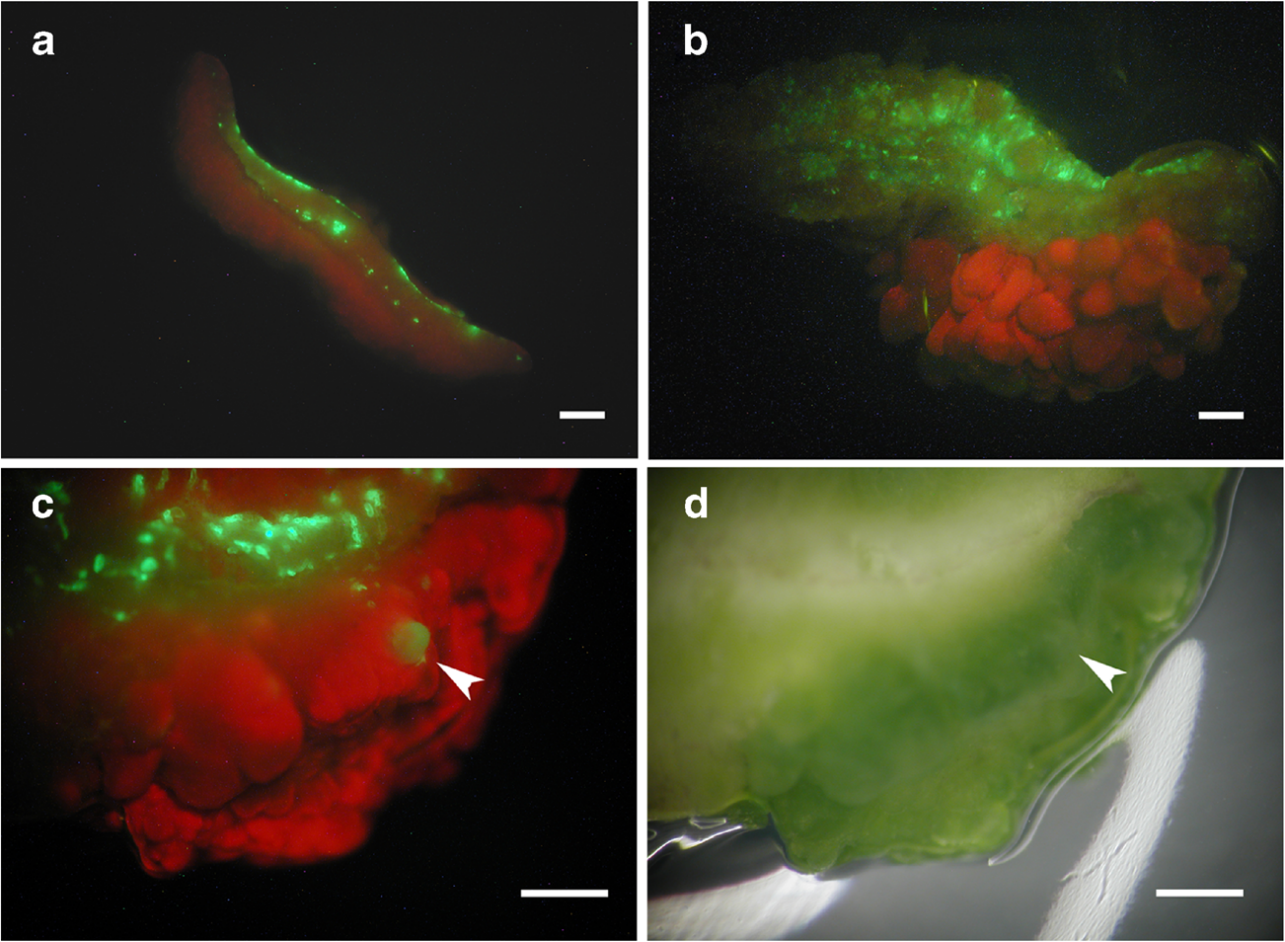
Transformation using high inoculum and 3-d co-culture followed by hygromycin B selection. (*a*) Explant showing the cut side at the top 8 d after inoculation. (*b*) Explant showing GFP in the cotyledon tissue with induced shoots on the adaxial side having no GFP expression 16 d after inoculation. (*c*), (*d*) Explant showing the cut side in contact with the medium 16 d after inoculation, with a single GFP shoot (*arrowhead*) and most GFP-expressing cells at the cut side; *c* under GFP excitation conditions (GFP-2 filter); *d* under brightfield (no filter). *Bar* = 1 mm.

Sunflower cotyledon tissues were susceptible to *Agrobacterium*-mediated transformation by EHA105, and transformed cells expressing GFP were observed as early as 2 d after inoculation, following use of high inoculum (OD_600_ between 0.5 and 0.6, approximately 10^8^–10^9^ CFU mL^−1^). Despite the strong GFP expression from the *HaUbi* promoter, most of the GFP-expressing cells were located at the cut sides of the cotyledons, where shoot organogenesis rarely occurred (Fig. 1*a*). In contrast, the cells on the adaxial side of cotyledon, where adventitious shoots mostly formed, rarely showed early GFP expression using high inoculum (Fig. 1*a*, *b*). In tobacco and maize cells, transgene expression was detected within 24 h after *A. tumefaciens* inoculation (Narasimhulu *et al.*^1996^), and a 2–3-d co-culture has traditionally been used for generating transformed cells and plants (Godwin *et al.*^1991^; Bidney *et al.*^1992^; Hiei *et al.*^1994^; Perl *et al.*^1996^; Trick and Finer 1997; Bond and Roose 1998; Zhao *et al.*^2002^; Cheng *et al.*^2004^; Ozawa 2009).

Although sunflower cotyledon explants displayed a high shoot production response, the frequency of GFP shoot production was very low with high *A. tumefaciens* inoculum levels and 3 d of co-culture followed by a hygromycin B selection (Fig. 1*b*–*d*). Explant preparation generated many wounded exposed cells at the cut side of the cotyledon, and these cells apparently produced the phenolic compounds and monosaccharides that are chemotactic and inducers of *A. tumefaciens* virulence genes (Parke *et al.*^1987^; Cangelosi *et al.*^1990^). With high inoculum levels, the large numbers of bacteria apparently transformed the cells located in the wounded tissues during the early co-culture period. Although transformation of wounded sunflower tissue does not appear to be difficult, the rapidly transformed cells in the cut regions do not necessarily contribute to shoot formation. The difficulty in targeting regeneration-competent cells has been one of the major challenges in producing transgenic sunflower plants (Laparra *et al.*^1995^).

### **LI**/**LC increased the frequency of GFP shoot production**

**Figure 2.**
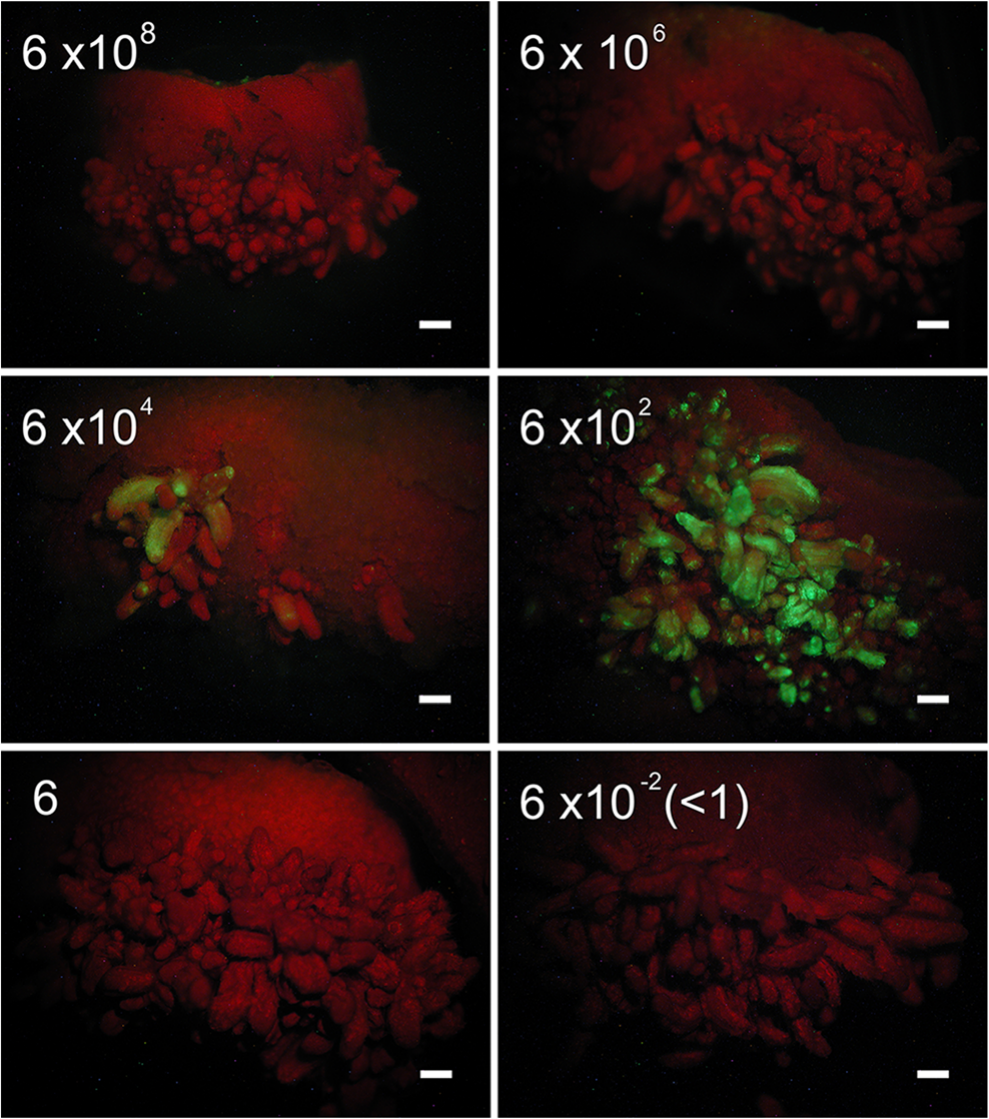
GFP-expressing shoots observed on cotyledon explants inoculated with *Agrobacterium* suspensions of either 6 × 10^8^, 6 × 10^6^, 6 × 10^4^, 6 × 10^2^, 6, or 6 × 10^−2^ CFU mL^−1^ (equivalent to undiluted, 10^−2^, 10^−4^, 10^−6^, 10^−8^, or 10^−10^ dilution, respectively) after 15-d co-culture. *Bar* = 1 mm.

**Figure 3.**
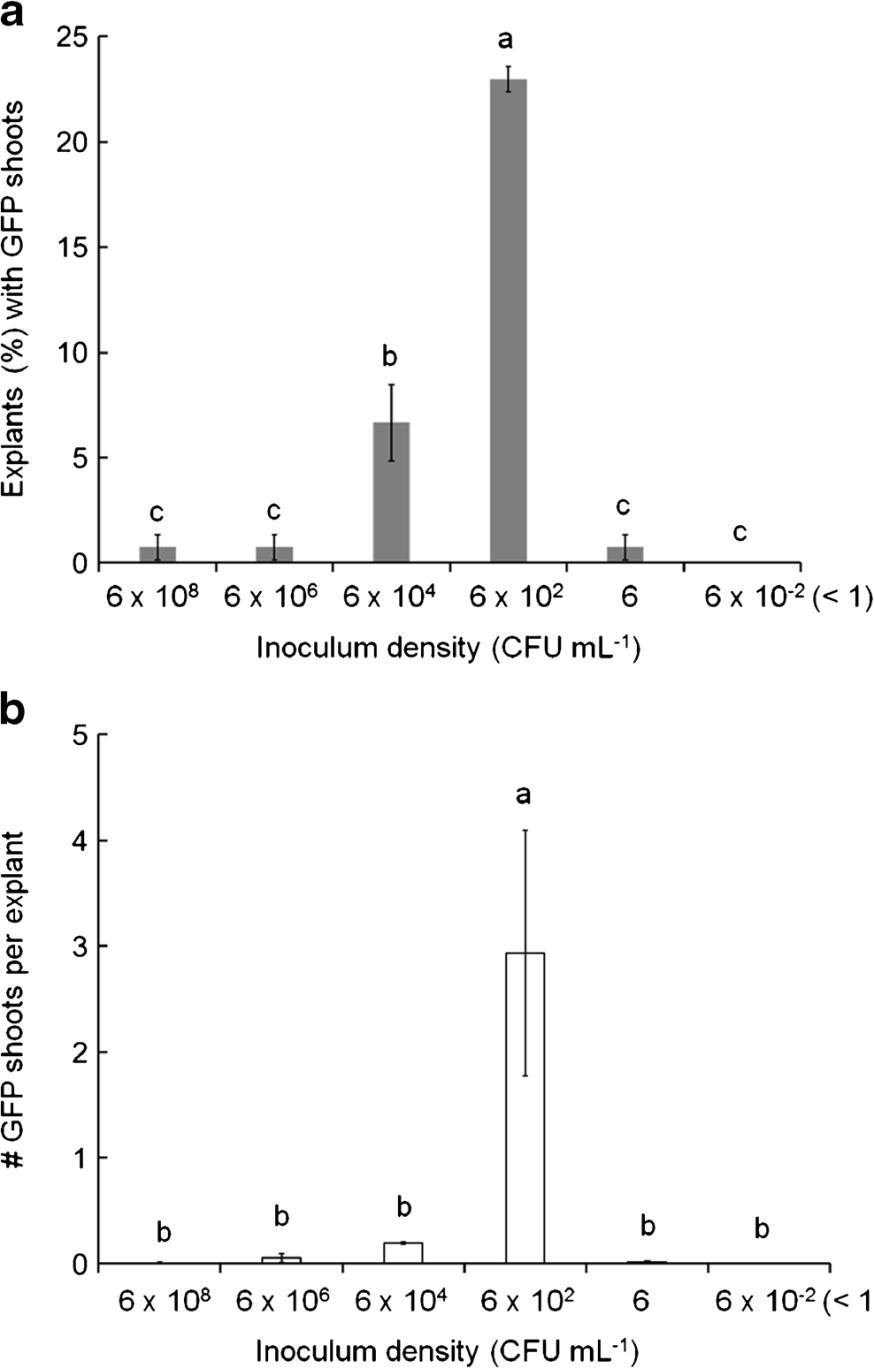
Transformation efficiency of cotyledon explants with inoculum suspensions of 6 × 10^8^, 6 × 10^6^, 6 × 10^4^, 6 × 10^2^, 6, or 6 × 10^−2^ CFU mL^−1^ (equivalent to undiluted, 10^−2^, 10^−4^, 10^−6^, 10^−8^, or 10^−10^ dilution, respectively) after 15 d of co-culture. (*a*) Mean percentages of explants having GFP shoots. (*b*) Means of GFP shoots per explant. *Bars* represent standard error, with different *letters* indicating significant difference based on Duncan’s multiple range test (*p* < 0.05). Values are mean ± standard error.

The use of low inoculum suspensions (approximately 6 × 10^2^ CFU mL^−1^) with 15-d-long co-culture resulted in the production of transformed cells at the shoot-forming regions with much higher efficiency, leading to a significantly higher number of GFP shoots than obtained using high inoculum (Fig. 2). The high inoculum (OD_600_ ≈ 0.55) contained about 6 × 10^8^ CFU mL^−1^, while its 10^−2^, 10^−4^, 10^−6^, 10^−8^, and 10^−10^ dilutions contained about 6 × 10^6^, 6 × 10^4^, 6 × 10^2^, 6, and 6 × 10^−2^ CFU mL^−1^, respectively. Unlike the high inoculum treatment, the use of diluted bacterial suspensions did not result in detectable early GFP expression in wounded tissues, probably due to the lower numbers of *A. tumefaciens* cells on each explant at the early time point. By the time the *A. tumefaciens* population increased, those early-wounded cells in the cut cotyledon may not have been as susceptible to *Agrobacterium*-mediated transformation, which might explain why the LI/LC method led to fewer transformed cells at the cut sides than high inoculum. Regardless, the use of a low inoculum suspension with about 6 × 10^2^ CFU mL^−1^ led to a significant increase in the production of GFP shoots after a 15-d-long co-culture (Fig. 2). More than 20% of the explants exposed to this low inoculum formed GFP shoots, with about three GFP shoots per explant (Fig. 3). When bacterial density in the *A. tumefaciens* suspension was either higher or lower than 6 × 10^2^ CFU mL^−1^, the percentage of explants with GFP shoots and the numbers of GFP shoots per explant were both lower (Fig. 3). Interaction between inoculum density and co-culture time was observed, and low inoculum and long co-culture were both required for the increased transformation efficiency of sunflower shoots (Table 4). Neither low inoculum with short co-culture nor high inoculum with either short or long co-culture yielded any GFP shoots (Table 4). In addition, the production of transformed shoots with the LI/LC method without hygromycin B selection was 30-fold higher than the traditional approach of using high inoculum/short co-culture plus Hyg-selection, where 6% explants formed GFP shoots, with 0.06 GFP shoots per explant (data not shown).

**Table 4.** The effect of inoculum density and co-culture time on sunflower transformation

Co-culture time (d)	Percentage of explants with GFP shoots (numbers of GFP shoots per explant)	
	*Agrobacterium* inoculum density (CFU mL^−1^)	
	6 × 10^8^	6 × 10^2^
3	0.0 ± 0.0 (0.0 ± 0.0) ^a^	0.0 ± 0.0 (0.0 ± 0.0) ^a^
15	0.0 ± 0.0 (0.0 ± 0.0) ^a^	23.7 ± 2.3 (1.8 ± 0.7) ^b^

Data represent mean ± standard error based on three replicates, each replicate containing 45 explants. Mean values followed by different *letters* are significantly different based on Duncan’s multiple range test (*p* < 0.05)

**Figure 4.**
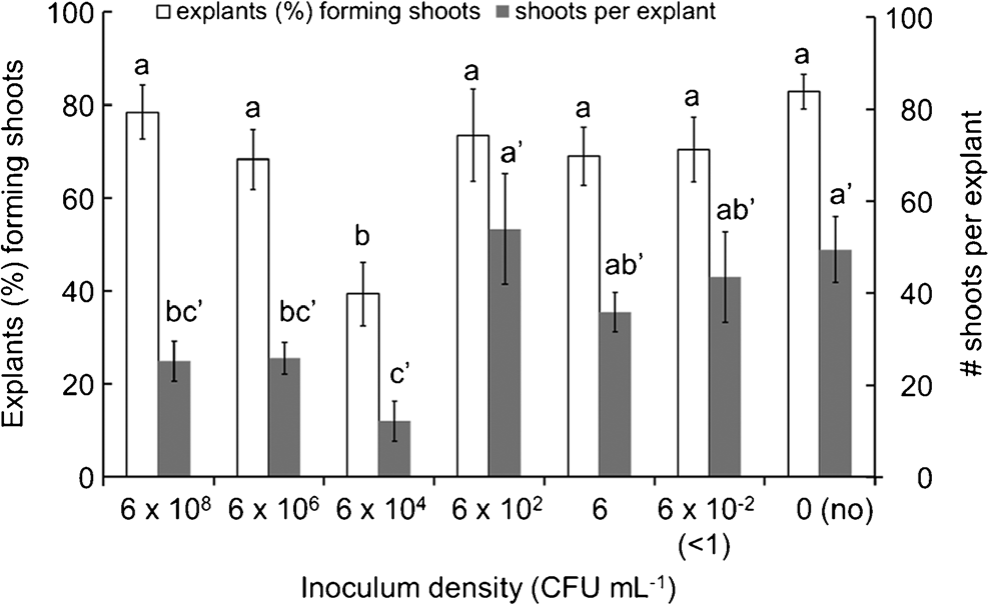
Shoot organogenesis of cotyledon explants after inoculation with *Agrobacterium* suspensions of 6 × 10^8^, 6 × 10^6^, 6 × 10^4^, 6 × 10^2^, 6, 6 × 10^−2^, or 0 (no inoculation, no) CFU mL^−1^ (equivalent to undiluted, 10^−2^, 10^−4^, 10^−6^, 10^−8^, 10^−10^ dilution, or negative control, respectively) followed by 15-d co-culture. Data represent the mean percentage of cotyledon explants forming shoots (*white*) and the mean of shoots per explant (*gray*) after 15 d of culture. *Bars* represent standard error, with different *letters* within each group (*white* or *gray*, no mark or prime, respectively) indicating significant difference based on Duncan’s multiple range test (*p* < 0.05). Values are mean ± standard error.

The increase of GFP shoot production by the LI/LC method could be attributable to the extended time of interaction between bacteria and plant tissues. With extended interaction, *A. tumefaciens* would likely have more opportunities to target and transform the rapidly growing cells that are involved in shoot formation and plant regeneration, producing more transgenic shoots. Previous attempts to increase transformation by extending co-culture period with high inoculum were unsuccessful, as plant regeneration were severely suppressed by bacterial overgrowth and no improvement of transgenic shoot production was observed (Rashid *et al.*^1996^; Cervera *et al.*^1998^; Sujatha *et al.*^2012^). In the present study, the number of shoots arising from explants treated with low inoculum was similar to the number produced without inoculation, and higher than those treated with high inoculum (Fig. 4). Although alternate wounding approaches have been employed for successful transformation of sunflower tissues (Bidney *et al.*^1992^; Grayburn and Vick 1995; Weber *et al.*^2003^), too much wounding also reduces the regeneration response due to disruptions to organized tissues (Trick and Finer 1997). With the use of LI/LC shown here, additional wounding was not necessary for the enhanced transformation in the shoot-forming regions, making the LI/LC method valuable for tissues where wounding is undesirable. It is still possible that moderate wounding or the application of other approaches could further expand the applications of the LI/LC approach.

### **Low inoculum led to lower infestation rates**

**Figure 5.**
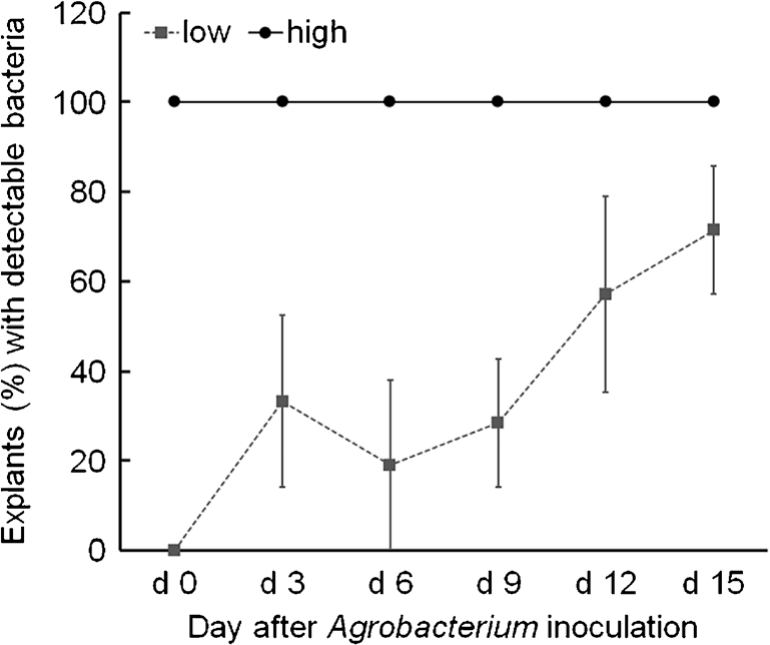
The change in the percentage of explants with detectable *Agrobacterium* during 15-d co-culture after inoculation of explants with either low inoculum (low) or high inoculum (high). Values are mean ± standard error.

At 0 d (immediately after inoculation), *A. tumefaciens* cells were detected on every explant treated with high inoculum, but not on any explants treated with low inoculum (Fig. 5). Bacteria were detected in some explants 3 d after the low inoculum treatment, but the percentage of explants with detectable bacteria never reached 100%. In contrast, bacteria were detected on all the explants sampled during 15 d of co-culture after the high inoculum treatment (Fig. 5). The presence of *A. tumefaciens* on explants did not necessarily lead to GFP shoots. With high inoculum, GFP shoots were rarely obtained, regardless of the consistent detection of *A. tumefaciens* cells. Bacteria were always detected on explants producing GFP shoots. With low inoculum, bacteria were detected on explants forming GFP shoots as well as those having no GFP shoots. High inoculum could lead to visible bacterial growth on the co-culture medium within 1 wk while low inoculum did not result in visible bacterial growth around explants until almost the end of the long co-culture period.

The large variation in the percentage of explants with detectable bacteria observed with the low inoculum treatment indicated some variation in the initial number of bacteria on each explant, which could explain the considerable variation of transgenic shoots arising from each explant (Fig. 3). Many explants that did not form any GFP shoots may simply not have been inoculated with a single *A. tumefaciens* cell or the inoculated bacteria did not survive. Using traditional inoculation methods, explants were dipped in and exposed to a bacterial suspension, but the number of bacteria that became attached to the explants could not be controlled. With an inoculum containing tenfold more bacteria than the low inoculum, about three viable bacteria on average were detected on each explant after inoculation (data not shown), suggesting that bacterial numbers on explants inoculated with low inoculum were very small, perhaps under the detection limit of the YEP plating assay. Given this situation, most of explants might not have been infected when using inocula containing less than 6 × 10^2^ CFU mL^−1^, so that diminished transformation was observed (Figs. 2 and 3).

To more precisely control the amount of inoculated bacteria and reduce some of the variability in the production of transformed shoots using the LI/LC approach, an alternate inoculation method of directly pipetting defined volumes of dilute bacterial suspensions was explored. Although more controlled inoculation of low numbers of bacteria was achieved, more consistent GFP shoot production among inoculated explants was not observed with this approach (data not shown). As the LI/LC method is further developed and optimized, other inoculation methods will likely lead to further increases in transformation efficiencies.

### **Low inoculum led to reduced expression of plant defense genes**

**Figure 6.**
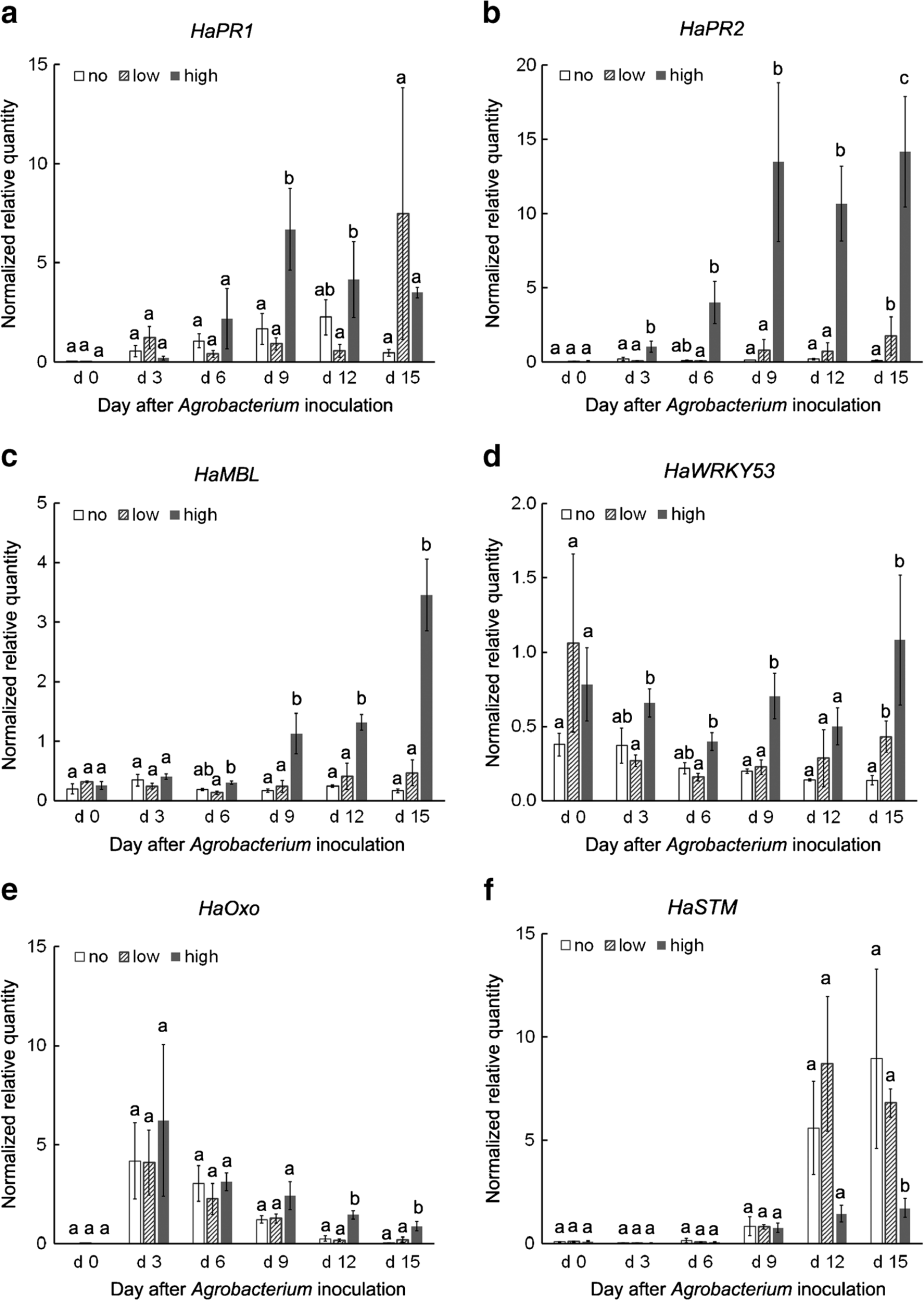
Relative expression levels of *HaPR1*, *HaPR2*, *HaMBL*, *HaWRKY53*, *HaOxo*, and *HaSTM* in explants inoculated with either no inoculation (no), low inoculum (low), or high inoculum (high) over 15 d of co-culture determined using qRT-PCR. Different *letters* indicate significant difference among treatments at the same time point based on Duncan’s multiple range test (*p* < 0.05).

Orthologs of five sunflower genes (Table 2) were identified based on five *A. thaliana* genes that were upregulated after *A. tumefaciens* infection and contributed to defense responses (Ditt *et al.*^2006^; Lee *et al.*^2009^). The bidirectional BLAST analysis confirmed that the sunflower genes and the corresponding *A. thaliana* genes belonged to the same gene family. Differential expression of these genes was observed between the low inoculum and high inoculum treatments. Based on qRT-PCR, their expression in explants treated with low inoculum was, in general, lower than with high inoculum (Fig. 6). The lower expression levels of some key genes associated with plant defense responses in the low inoculum treatment suggested that a reduction in expression of plant defense response genes may reduce or eliminate the inhibition of *Agrobacterium*-mediated plant transformation (Veena *et al.*^2003^; Zipfel *et al.*^2006^).

A sunflower ortholog of PR protein 1 (*HaPR1*) was expressed at higher levels under high inoculum than low inoculum at 9 and 12 d (Fig. 6*a*). *HaPR1* expression as 9 d after the high inoculum treatment was sevenfold higher than the low inoculum treatment, while its expression after the low inoculum treatment remained low until the end of long co-culture (Fig. 6*a*). After 3 d of co-culture, a sunflower ortholog of PR protein 2 (*HaPR2*) was expressed 20-fold higher in explants treated with high inoculum than low inoculum, and its expression continued to increase (Fig. 6*b*). *HaPR2* expression in tissues treated with high inoculum was consistently over tenfold higher than in tissues treated with low inoculum during co-culture from 3 to 15 d (Fig. 6*b*). Since the accumulation of PR proteins is associated with systematic acquired resistance (SAR) (Ward *et al.*^1991^; Uknes *et al.*^1992^), the higher expression of PR proteins in the high inoculum treatment indicated the activation of SAR. The induction of SAR with high inoculum may have contributed to reduced transformation rates in sunflower, as constitutive expression of PR proteins has previously been shown to confer resistance to *Agrobacterium*-mediated transformation in *A. thaliana* (Veena *et al.*^2003^; Gaspar *et al.*^2004^). In contrast, the use of low inoculum probably delayed or avoided the activation of SAR, leading to enhanced transformation.

A bulb-type lectin/S-locus glycoprotein/mannose-binding lectin (*HaMBL*) also expressed at higher levels in the explants treated with high inoculum than those treated with low inoculum from 6 to 15 d during co-culture (Fig. 6*c*). *HaMBL* expression in explants after high inoculum treatment continued to increase while its expression in explants treated with low inoculum remained almost unchanged (Fig. 6*c*). Since mannose-binding lectin/bulb-type lectins are potential receptors of lipopolysaccharides that are the major components of the bacterial outer membrane, and thus may serve as PAMP signals (Dow *et al.*^2000^), induction of a mannose-binding lectin gene *HaMBL* by high inoculum suggests the activation of PTI that could inhibit *Agrobacterium*-mediated transformation. Bulb-type lectin genes have been associated with plant defense responses to bacterial pathogens in *A. thaliana* (Ranf *et al.*^2015^) and pepper (*Capsicum annuum*, Hwang and Hwang 2011). Although a direct link between the bulb-type lectin/mannose-binding lectin and resistance to *A. tumefaciens* has not been clearly demonstrated, induction of expression of the *HaMBL* gene in this study as well as previous gene expression results from inoculating *A. thaliana* inflorescence stalks with *A. tumefaciens* (Lee *et al.*^2009^) suggest that it functions as a receptor for some unknown PAMP signals (Zipfel *et al.*^2006^). Accumulated PAMP signals probably activate plant defense responses that result in lower transformation efficiencies (Zipfel *et al.*^2006^).

High inoculum led to slightly higher expression of *WRKY53* than low inoculum. High expression of *HaWRKY53* was observed soon after explant preparation (0 d, Fig. 6*d*), yet there was no significant difference among inoculation treatments. After 3 d of co-culture, the expression of *HaWRKY53* started to decline in explants treated with no inoculation and low inoculum, while its expression in explants treated with high inoculum remained stable, being about twofold higher than in explants treated with low inoculum from 3 to 9 d (Fig. 6*d*). *HaWRKY53* expression in low inoculum increased and reached a level comparable to high inoculum at 12 and 15 d. Transcription factor *WRKY53* is a marker for the early stages of senescence (Hinderhofer and Zentgraf 2001) and is also associated with stress responses (Miao *et al.*^2004^). The relatively high expression of *HaWRKY53* at 0 d could be attributable to the wounds generated during explant preparation. Higher expression of *HaWRKY53* in explants treated with high inoculum from 3 to 9 d indicated that explants underwent more severe stress or more cells underwent HR than with low inoculum during this period, which could limit the transformation of the induced cells that have the potential to contribute to meristem formation and shoot formation.

An oxoglutarate/iron-dependent dioxygenase gene (*HaOxo*) expressed at a higher level with the high inoculum treatment than with the low inoculum treatment at 12 and 15 d during co-culture. Although no difference was observed among treatments from 0 to 9 d, its expression in the high inoculum treatment was over sevenfold higher than in the low inoculum treatment during the later stage of co-culture (Fig. 6*e*). Since the *A. thaliana* ortholog of *HaOxo* is associated with PCD induced by H_2_O_2_ (Gechev *et al.*^2005^) and also induced by *A. tumefaciens* infection (Lee *et al.*^2009^), the higher expression of *HaOxo* at the later stage of co-culture with high inoculum suggested that more plant cells were progressing through PCD at this time point. After exposure to large numbers of *A. tumefaciens* cells, plant defense was likely induced, probably leading to apoplasmic alkalization and reactive oxidative burst that could function as apoptosis signals (Mur *et al.*^2008^) and negatively affect plant regeneration from transformed cells.

In addition, the expression of the sunflower ortholog of the *Arabidopsis shoot meristemless* (*STM*) gene (*HaSTM*) that is related to shoot meristem formation, tended to be higher with the low inoculum treatment than the high inoculum treatment. A significant difference was observed at 15 d between the low inoculum and the high inoculum treatments (Fig. 6*f*). The higher expression of *HaSTM* in the low inoculum treatment suggested higher shoot meristem activity in the explants treated with low inoculum than high inoculum. Higher shoot induction responses were observed with the low inoculum treatment compared to the high inoculum treatment (Fig. 2). A high shoot induction response likely contributed to a higher production of transformed shoots, when low inoculum was used with long co-culture.

## Conclusions

A novel *Agrobacterium*-mediated transformation procedure was developed for sunflower, using low inoculum at about 6 × 10^2^ CFU mL^−1^ with a long co-culture period of 15 d. With high inoculum and short (2–3 d) co-culture, the single cells that were transformed were located in freshly cut tissues. These transformed cells apparently did not contribute to later plant regeneration. In contrast, use of low inoculum with a long co-culture period increased the chances of transforming cells that contributed to meristem formation. The use of low inoculum allowed an extension of co-culture time, increasing the opportunity for *A. tumefaciens* to interact with appropriate target cells. As opposed to high inoculum, use of low inoculum levels did not lead to suppression of plant regeneration or activation of defense responses. The use of LI/LC may be more similar to the infestation of plants by *Agrobacterium* sp. in nature, where relatively low amounts of bacteria are found in the soil (Benzle *et al.*^2015^). The approach described here could lead to improvements in transformation efficiencies of other plants and development of targets, which have not previously been considered useful for transformation.

## References

[CR1] Alt-Mörbe J, Kühlmann H, Schröder J (1989). Differences in induction of Ti plasmid virulence genes *virG* and *virD*, and continued control of *virD* expression by four external factors. Mol Plant Microbe Interact.

[CR2] Benzle KA, Finer KR, Marty D, McHale LK, Goodner BW, Taylor CG, Finer JJ (2015). Isolation and characterization of novel *Agrobacterium* strains for soybean and sunflower transformation. Plant Cell Tissue Organ Cult.

[CR3] Bevan MW, Flavell RB, Chilton M (1983). A chimaeric antibiotic resistance gene as a selectable marker for plant cell transformation. Nature.

[CR4] Bidney D, Scelonge C, Martich J, Burrus M, Sims L, Huffman G (1992). Microprojectile bombardment of plant tissues increases transformation frequency by *Agrobacterium tumefaciens*. Plant Mol Biol.

[CR5] Blanc G, Baptiste C, Oliver G, Martin F, Montoro P (2006). Efficient *Agrobacterium tumefaciens*-mediated transformation of embryogenic calli and regeneration of *Hevea brasiliensis* Müll Arg. plants. Plant Cell Rep.

[CR6] Bond JE, Roose ML (1998). *Agrobacterium*-mediated transformation of the commercially important citrus cultivar Washington navel orange. Plant Cell Rep.

[CR7] Cangelosi GA, Ankenbauer RG, Nester EW (1990). Sugars induce the *Agrobacterium* virulence genes through a periplasmic binding protein and a transmembrane signal protein. Proc Natl Acad Sci U S A.

[CR8] Cervera M, Pina J, Juarez J, Navarro L, Pena L (1998). *Agrobacterium*-mediated transformation of citrange: factors affecting transformation and regeneration. Plant Cell Rep.

[CR9] Chen H, Nelson RS, Sherwood JL (1994). Enhanced recovery of transformants of *Agrobacterium tumefaciens* after freeze-thaw transformation and drug selection. Biotechniques.

[CR10] Cheng M, Hu T, Layton J, Liu CN, Fry JE (2003). Desiccation of plant tissues post-*Agrobacterium* infection enhances T-DNA delivery and increases stable transformation efficiency in wheat. In Vitro Cell Dev Biol Plant.

[CR11] Cheng M, Lowe BA, Spencer TM, Ye X, Armstrong CL (2004). Factors influencing *Agrobacterium*-mediated transformation of monocotyledonous species. In Vitro Cell Dev Biol Plant.

[CR12] Clough SJ, Bent AF (1998). Floral dip: a simplified method for *Agrobacterium*-mediated transformation of *Arabidopsis thaliana*. Plant J.

[CR13] Coll NS, Epple P, Dangl JL (2011). Programmed cell death in the plant immune system. Cell Death Differ.

[CR14] de Ropp RS (1947). The growth-promoting and tumefacient factors of bacteria-free crown-gall tumor tissue. Am J Bot.

[CR15] Ditt RF, Kerr KF, de Figueiredo P, Delrow J, Comai L, Nester EW (2006). The *Arabidopsis thaliana* transcriptome in response to *Agrobacterium tumefaciens*. Mol Plant Microbe Interact.

[CR16] Dow M, Newman MA, von Roepenack E (2000). The induction and modulation of plant defense responses by bacterial lipopolysaccharides. Annu Rev Phytopathol.

[CR17] Everett NP, Robinson KEP, Mascarenhas D (1987). Genetic engineering of sunflower (Helianthus annuus L.). Nat Biotechnol.

[CR18] Fillatti JJ, Sellmer J, McCown B, Haissig B, Comai L (1987). *Agrobacterium* mediated transformation and regeneration of *Populus*. Mol Gen Genet.

[CR19] Fraley RT, Rogers SG, Horsch RB, Sanders PR, Flick JS, Adams SP, Bittner ML, Brand LA, Fink CL, Fry JS, Galluppi GR, Goldberg SB, Hoffmann NL, Woo SC (1983). Expression of bacterial genes in plant cells. Proc Natl Acad Sci U S A.

[CR20] Frame BR, Shou H, Chikwamba RK, Zhang Z, Xiang C, Fonger TM, Pegg SEK, Li B, Nettleton DS, Pei D, Wang K (2002). *Agrobacterium tumefaciens*-mediated transformation of maize embryos using a standard binary vector system. Plant Physiol.

[CR21] Gamborg OL, Miller R, Ojima K (1968). Nutrient requirements of suspension cultures of soybean root cells. Exp Cell Res.

[CR22] Gaspar YM, Nam J, Schultz CJ, Lee LY, Gilson PR, Gelvin SB, Bacic A (2004). Characterization of the *Arabidopsis* lysine-rich arabinogalactan-protein *AtAGP17* mutant (*rat1*) that results in a decreased efficiency of *Agrobacterium transformation*. Plant Physiol.

[CR23] Gechev T, Minkov I, Hille J (2005). Hydrogen peroxide induced cell death in *Arabidopsis*: transcriptional and mutant analysis reveals a role of an oxoglutarate dependent dioxygenase gene in the cell death process. IUBMB Life.

[CR24] Gelvin SB (2003). *Agrobacterium*-mediated plant transformation: the biology behind the “gene-jockeying” tool. Microbiol Mol Biol Rev.

[CR25] Gelvin SB (2012). Traversing the cell: *Agrobacterium* T-DNA’s journey to the host genome. Front Plant Sci.

[CR26] Godwin I, Todd G, Ford-Lloyd B, Newbury HJ (1991). The effects of acetosyringone and pH on *Agrobacterium*-mediated transformation vary according to plant species. Plant Cell Rep.

[CR27] Grayburn WS, Vick BA (1995). Transformation of sunflower (*Helianthus annuus* L.) following wounding with glass beads. Plant Cell Rep.

[CR28] Hansen G (2000). Evidence for *Agrobacterium*-induced apoptosis in maize cells. Mol Plant Microbe Interact.

[CR29] Hansen G, Das A, Chilton MD (1994). Constitutive expression of the virulence genes improves the efficiency of plant transformation by *Agrobacterium*. Proc Natl Acad Sci U S A.

[CR30] Hellemans J, Mortier G, De Paepe A, Speleman F, Vandesompele J (2007). qBase relative quantification framework and software for management and automated analysis of real-time quantitative PCR data. Genome Biol.

[CR31] Herrera-Estrella L, Block MD, Messens E, Hernalsteens JP, Montagu MV, Schell J (1983). Chimeric genes as dominant selectable markers in plant cells. EMBO J.

[CR32] Hiei Y, Ohta S, Komari T, Kumashiro T (1994). Efficient transformation of rice (*Oryza sativa* L.) mediated by *Agrobacterium* and sequence analysis of the boundaries of the T-DNA. Plant J.

[CR33] Hinderhofer K, Zentgraf U (2001). Identification of a transcription factor specifically expressed at the onset of leaf senescence. Planta.

[CR34] Hood EE, Gelvin SB, Melchers LS, Hoekema A (1993). New *Agrobacterium* helper plasmids for gene transfer to plants. Transgenic Res.

[CR35] Hwang IS, Hwang BK (2011). The pepper mannose-binding lectin gene *CaMBL1* is required to regulate cell death and defense responses to microbial pathogens. Plant Physiol.

[CR36] Jones JD, Dangl JL (2006). The plant immune system. Nature.

[CR37] Lamb C, Dixon RA (1997). The oxidative burst in plant disease resistance. Annu Rev Plant Physiol Plant Mol Biol.

[CR38] Laparra H, Burrus M, Hunold R, Damm B, Bravo-Angel AM, Bronner R, Hahne G (1995). Expression of foreign genes in sunflower (*Helianthus annuus* L.)—evaluation of three gene transfer methods. Euphytica.

[CR39] Lee CW, Efetova M, Engelmann JC, Kramell R, Wasternack C, Ludwig-Müller J, Hedrich R, Deeken R (2009). *Agrobacterium tumefaciens* promotes tumor induction by modulating pathogen defense in *Arabidopsis thaliana*. Plant Cell.

[CR40] Leeman M, van Pelt JA, den Ouden FM, Heinsbroek M, Bakker PAHM, Schippers B (1995). Induction of systemic resistance by *Pseudomonas fluorescens* in radish cultivars differing in susceptibility to fusarium wilt, using a novel bioassay. Eur J Plant Pathol.

[CR41] Miao Y, Laun T, Zimmermann P, Zentgraf U (2004). Targets of the WRKY53 transcription factor and its role during leaf senescence in *Arabidopsis*. Plant Mol Biol.

[CR42] Mur LA, Kenton P, Lloyd AJ, Ougham H, Prats E (2008). The hypersensitive response; the centenary is upon us but how much do we know?. J Exp Bot.

[CR43] Murai N, Kemp JD, Sutton DW, Murray MG, Slightom JL, Merlo DJ, Reichert NA, Sengupta-Gopalan C, Stock CA, Barker RF, Hall TC (1983). Phaseolin gene from bean is expressed after transfer to sunflower via tumor-inducing plasmid vectors. Science.

[CR44] Murashige T, Skoog F (1962). A revised medium for rapid growth and bio assays with tobacco tissue cultures. Physiol Plant.

[CR45] Narasimhulu SB, Deng XB, Sarria R, Gelvin SB (1996). Early transcription of *Agrobacterium* T-DNA genes in tobacco and maize. Plant Cell.

[CR46] Olhoft P, Somers D (2001). L-cysteine increases *Agrobacterium*-mediated T-DNA delivery into soybean cotyledonary-node cells. Plant Cell Rep.

[CR47] Ozawa K (2009). Establishment of a high efficiency *Agrobacterium*-mediated transformation system of rice (*Oryza sativa* L.). Plant Sci.

[CR48] Parke D, Ornston LN, Nester EW (1987). Chemotaxis to plant phenolic inducers of virulence genes is constitutively expressed in the absence of the Ti plasmid in *Agrobacterium tumefaciens*. J Bacteriol.

[CR49] Perl A, Lotan O, Abu-Abied M, Holland D (1996). Establishment of an *Agrobacterium*-mediated transformation system for grape (*Vitis vinifera* L.): the role of antioxidants during grape-*Agrobacterium* interactions. Nat Biotechnol.

[CR50] Power CJ (1987). Organogenesis from *Helianthus annuus* inbreds and hybrids from the cotyledons of zygotic embryos. Am J Bot.

[CR51] Raaijmakers JM, Leeman M, van Oorschot MM, van der Sluis I, Schippers B, Bakker PAHM (1995). Dose–response relationships in biological control of fusarium wilt of radish by *Pseudomonas spp*. Phytopathology.

[CR52] Ranf S, Gisch N, Schäffer M, Illig T, Westphal L, Knirel YA, Sánchez-Carballo PM, Zähringer U, Hückelhoven R, Lee J, Scheel D (2015). A lectin S-domain receptor kinase mediates lipopolysaccharide sensing in *Arabidopsis thaliana*. Nat Immunol.

[CR53] Rashid H, Yokoi S, Toriyama K, Hinata K (1996). Transgenic plant production mediated by *Agrobacterium* in indica rice. Plant Cell Rep.

[CR54] Rieu I, Powers SJ (2009). Real-time quantitative RT-PCR: design, calculations, and statistics. Plant Cell.

[CR55] Santarém ER, Trick HN, Essig JS, Finer JJ (1998). Sonication-assisted *Agrobacterium*-mediated transformation of soybean immature cotyledons: optimization of transient expression. Plant Cell Rep.

[CR56] Sujatha M, Vijay S, Vasavi S, Reddy PV, Rao SC (2012). *Agrobacterium*-mediated transformation of cotyledons of mature seeds of multiple genotypes of sunflower (*Helianthus annuus* L.). Plant Cell Tissue Organ Cult.

[CR57] Trick HN, Finer JJ (1997). SAAT: Sonication-assisted *Agrobacterium*-mediated transformation. Transgenic Res.

[CR58] Uknes S, Mauch-Mani B, Moyer M, Potter S, Williams S, Dincher S, Chandler D, Slusarenko A, Ward E, Ryals J (1992). Acquired resistance in *Arabidopsis*. Plant Cell.

[CR59] Vandesompele J, De Preter K, Pattyn F, Poppe B, Van Roy N, De Paepe A, Speleman F (2002). Accurate normalization of real-time quantitative RT-PCR data by geometric averaging of multiple internal control genes. Genome Biol.

[CR60] Veena, Jiang H, Doerge RW, Gelvin SB (2003). Transfer of T-DNA and Vir proteins to plant cells by *Agrobacterium tumefaciens* induces expression of host genes involved in mediating transformation and suppresses host defense gene expression. Plant J.

[CR61] Ward ER, Uknes SJ, Williams SC, Dincher SS, Wiederhold DL, Alexander DC, Ahl-Goy P, Métraux JP, Ryals JA (1991). Coordinate gene activity in response to agents that induce systemic acquired resistance. Plant Cell.

[CR62] Weber S, Friedt W, Landes N, Molinier J, Himber C, Rousselin P, Hahne G, Horn R (2003). Improved *Agrobacterium*-mediated transformation of sunflower (*Helianthus annuus* L.): assessment of macerating enzymes and sonication. Plant Cell Rep.

[CR63] Zhang Z, Finer JJ (2015). Sunflower (*Helianthus annuus* L.) organogenesis from primary leaves of young seedlings preconditioned by cytokinin. Plant Cell Tissue Organ Cult.

[CR64] Zhao Z, Gu W, Cai T, Tagliani L, Hondred D, Bond D, Schroeder S, Rudert M, Pierce D (2002). High throughput genetic transformation mediated by *Agrobacterium tumefaciens* in maize. Mol Breed.

[CR65] Zhou X, Wang K, Lv D, Wu C, Li J, Zhao P, Ye X (2013). Global Analysis of differentially expressed genes and proteins in the wheat callus infected by *Agrobacterium tumefaciens*. PLoS One.

[CR66] Zipfel C, Kunze G, Chinchilla D, Caniard A, Jones JD, Boller T, Felix G (2006). Perception of the bacterial PAMP EF-Tu by the receptor EFR restricts *Agrobacterium*-mediated transformation. Cell.

